# A conversation with James Zou, PhD, Assistant Professor of Biomedical Data Science, Stanford University

**DOI:** 10.1017/cts.2022.487

**Published:** 2022-12-27

**Authors:** Kathy Siranosian

**Affiliations:** Clinical Research Forum, Washington, DC, USA

## Top 10 Clinical Research Achievement Awards Q & A

This article is part of a series of interviews with recipients of Clinical Research Forum’s Top 10 Clinical Research Achievement Awards. This article is with James Zou, PhD, Assistant Professor of Biomedical Data Science, Stanford University. Dr. Zou and his colleagues developed Trial Pathfinder, an open-source software that can integrate real-world data and analyze the hazard ratio of the overall survival for cohorts defined by different eligibility criteria. Dr. Zou received a 2022 Top 10 Clinical Research Achievement Award for Evaluating eligibility criteria of oncology trials using real-world data and AI [1]*. The interview has been edited for length and clarity.*

## When Did You First Become Interested in Clinical Research?

I’m a computer scientist, and in the past, my work focused mostly on developing AI tools and machine learning models for genomics and basic biological discoveries. Over the past few years, I’ve become increasingly interested in how AI systems can be used for a variety of clinical research questions. The translational aspect is where I think AI can have the biggest real-world impact and already, we’re starting to see some exciting advances in this area. It’s incredibly motivating to be involved with not just developing algorithms, but also translating them into practice for the direct benefit of patients, clinicians, and whole healthcare systems.

## Your Paper is About Designing Eligibility Criteria of Oncology Trials. Was There a Reason You Focused on Oncology?

Oncology is one area where there’s now a lot of very well-curated real-world patient data. This includes structured and unstructured data from medical records and other sources, such as information related to diagnoses, genomic sequencing, previous treatments, and more. Over the last few years, there have been focused efforts to create and organize these very high-quality longitudinal oncology datasets.

## And These Data Sets Allow You to Computationally Emulate Clinical Trials?

Yes, exactly. Combining the large, real-world cohorts derived from the clinical records and the algorithm was the key to running the computational clinical trial. Actual clinical trials are resource-intensive. They can be extremely expensive, costing tens or maybe hundreds of millions of dollars, and take years and years to run. Running computational clinical trials is much cheaper and faster, and we can use the insights from them to inform the design of the real ones.

## Are There Other Advances That Have Enabled Computational Clinical Trials?

There have been several relatively recent developments that made our project possible. First, as I mentioned, on the data side, it’s only been quite recently that we’ve started to have well-curated, large patient cohorts – on the order of hundreds of thousands of cancer patients. Then, on the algorithm slide, we’ve had to overcome a lot of computational barriers to get these large-scale computations to emulate a trial in silico.

## What Else Makes This Research So Unique?

One of the most interesting aspects of our research is that it involves a very interdisciplinary team. As a computer scientist doing clinical research, I had to collaborate closely with others who were experienced in conducting clinical trials and working with large real-world cancer cohorts. While we all benefited from our complementary expertise, we also had to understand that we were all coming from different backgrounds, were using different terminologies, and had different ways of thinking about problems. One of our first steps was to come to an agreement about which problems were most important to solve. With clinical trials, the most important problems from a computer science or from a machine learning perspective might be very different from what’s most important from a clinical perspective.

## Is That How You Decided to Focus on Eligibility Requirements?

As it turns out, one of the biggest takeaways, one of the biggest “a-ha” moments, was when we realized that the most important problem from the clinical trial perspective is figuring out eligibility criteria for patients. That’s not something that I had considered before starting the project. But after having multiple in-depth discussions with our collaborators, we relatively quickly homed in on eligibility as the crux of the problem. The issue is that critical trials, and especially cancer critical trials, are currently overly restrictive, and that creates a number of different challenges. First, there are many patients who would like to participate in clinical trials – because that’s how they can get access to some of the most advanced lifesaving treatments – but they can’t because the trials are too restrictive and they’re not eligible. Then, that makes it harder for the drug developers and the pharma companies to enroll patients and run trials. It also creates big challenges for the rest of us because when these trials are so restrictive, the results may not be generalized. They might not reflect how the drug treatments work on patients in the general population.

## What Advice Do You Have for Computer Scientists or Others Who are Considering Getting More Involved with Clinical Research?

The best advice I have is to find good clinical partners from the very beginning. I have benefited tremendously from having strong partners – my colleagues at Genentech and many others – and I have learned so much from them. The MDs and MD-PhDs we work with see patients and also collaborate with us and having these close collaborations with the clinical side is so instrumental because it adds an entirely new perspective. Collaborations between disciplines are what’s going to have the most impact. The field of clinical research now involves large amounts of data, and it’s all of different types – imaging, medical records, genomics, etc. There’s really been a paradigm shift, and the research is becoming more and more data-driven. That’s why it’s increasingly critical for computer scientists and clinical researchers to work together.

## Is That What Motivates You to Continue Doing Clinical Research?

One big motivator is having great people to work with. I really enjoy working with the graduate students and postdocs in my lab and with my collaborators. We’re learning new things every day and having a lot of fun working on these projects together. The other big thing that keeps me motivated is keeping my eye on the big picture and staying focused on the impact of this research. Our findings from this study may help relax restrictive eligibility criteria for clinical trials. That will help make clinical trials more efficient, more representative, and more reliable. Those are very compelling end goals.

## Where is Your Research Headed?

I’m very excited about extending this *in silico* trial method to other diseases and I’m working on that now. I’m also very excited about expanding the capabilities of the algorithm within oncology. In follow-up work that was recently published, *Systematic pan-cancer analysis of mutation-treatment interactions using large real-world clinicogenomics data*, we show that we can actually take similar computational ideas for working with large, real-world oncology cohorts to discover predictive biomarkers for immunotherapy and targeted therapy [2]. We discovered over 400 of these quite strong predictive biomarkers which can help inform treatment recommendations in oncology. My team has been focused on oncology clinical trials, but we think that the methodology that we developed can be extended to other disease areas, as well. It can help people get the individualized treatment that works best for them.
